# Supplementary material for: Naturalistic development of trait mindfulness: A longitudinal examination of victimization and supportive relationships in early adolescence
Source: PLoS One. 2021 May 7;16(5):e0250960. doi: 10.1371/journal.pone.0250960 (PMC8104379; doi:10.1371/journal.pone.0250960)
Supplement: S1 Appendix — (DOCX) [file pone.0250960.s001.docx]

**S1 Appendix: Complete List of Items for All Constructs**

**Trait mindfulness (AIR & CASEL, 2013)**

1. When I’m upset, I notice how I am feeling before I take action.
2. When difficult situations happen, I can pause without immediately acting.
3. I am aware of how my moods affect the way I treat other people.

Response scale: 1 = *Disagree a lot*, 2 = *Disagree a little*, 3 = *Don’t agree or disagree*, 4 = *Agree a little*, 5 = *Agree a lot*

**Peer victimization (Law et al., 2012; Trach et al., 2010)**

*Important definition:* **Bully** – There are a lot of different ways to bully someone, but a bully has some advantage (stronger, more popular, or something else), wants to hurt the other person (it’s not an accident), and does so repeatedly (over and over again) and unfairly. Sometimes a group of students will bully another student.

This school year, how often have you been bullied by other students in the following ways?

1. Physical bullying (for example, someone hit, shoved, or kicked you, spat at you, beat you up, or damaged or took your things without permission).
2. Verbal bullying (for example, someone called you names, teased, embarrassed, threatened you, or made you do things you didn’t want to do).
3. Social bullying (for example, someone left you out, excluded you, gossiped and spread rumors about you, or made you look foolish).
4. Cyberbullying (for example, someone used the computer or text messages to exclude, threaten, embarrass you, or to hurt your feelings).

Response scale: 1 = *Not at all this school year*, 2 = *Once or a few times*, 3 = *About every month*, 4 = *About every week*, 5 = *Many times a week*

**Connectedness with adults at home (Constantine & Benard, 2001)**

In my home, there is a parent or another adult …

1. … who believes that I will be a success.
2. … who listens to me when I have something to say.
3. … who I can talk to about my problems.

Response scale: 1 = *Not at all true*, 2 = *A little true*, 3 = *Pretty much true*, 4 = *Very much true*

**Peer belonging (Hayden-Thomson, 1989)**

1. I feel part of a group of friends that do things together.
2. I feel that I usually fit in with other kids around me.
3. When I am with other kids my age, I feel I belong.

Response scale: 1 = *Disagree a lot*, 2 = *Disagree a little*, 3 = *Don’t agree or disagree*, 4 = *Agree a little*, 5 = *Agree a lot*

**Long-term self-regulation (Moilanen, 2007)**

1. If something isn’t going according to my plans, I change my actions to try and reach my goal.
2. When I have a serious disagreement with someone, I can talk calmly about it without losing control.
3. I work carefully when I know something will be tricky.

Response scale: 1 = *Disagree a lot*, 2 = *Disagree a little*, 3 = *Don’t agree or disagree*, 4 = *Agree a little*, 5 = *Agree a lot*
