# Supplementary material for: Naturalistic development of trait mindfulness: A longitudinal examination of victimization and supportive relationships in early adolescence
Source: PLoS One. 2021 May 7;16(5):e0250960. doi: 10.1371/journal.pone.0250960 (PMC8104379; doi:10.1371/journal.pone.0250960)
Supplement: S2 Appendix — (DOCX) [file pone.0250960.s002.docx]

**S2 Appendix: M*plus* Scripts**

Provided below are selected M*plus* scripts for key analyses.

**Variable centered analyses:**

**Cross-Sectional Measurement Model (7^th^ Grade)**

Variable: names are Cohort School Gender

SRG4 SRG4_1 SRG4_2 SRG4_3

SRG7 SRG7_1 SRG7_2 SRG7_3

ViG4 ViG4_1 ViG4_2 ViG4_3 ViG4_4

PBG4 PBG4_1 PBG4_2 PBG4_3

AHG4 AHG4_1 AHG4_2 AHG4_3

ViG7 ViG7_1 ViG7_2 ViG7_3 ViG7_4

PBG7 PBG7_1 PBG7_2 PBG7_3

AHG7 AHG7_1 AHG7_2 AHG7_3

MiG7 MiG7_1 MiG7_2 MiG7_3;

usevariables are

ViG7_1 ViG7_2 ViG7_3 ViG7_4

AHG7_1 AHG7_2 AHG7_3

PBG7_1 PBG7_2 PBG7_3

MiG7_1 MiG7_2 MiG7_3;

missing all (999);

Analysis: estimator = MLR;

Model: VictL by ViG7_1* ViG7_2 ViG7_3 ViG7_4;

AdultsL by AHG7_1* AHG7_2 AHG7_3;

PeersL by PBG7_1* PBG7_2 PBG7_3;

MindL by MiG7_1* MiG7_2 MiG7_3;

!Fixed factor method (fix latent factor variances !to 1)

VictL@1 AdultsL@1 PeersL@1 MindL@1;

OUTPUT: modindices (all) stdyx;

**Cross-Sectional Structural Model (7^th^ Grade)**

Variable: names are Cohort School Gender

SRG4 SRG4_1 SRG4_2 SRG4_3

SRG7 SRG7_1 SRG7_2 SRG7_3

ViG4 ViG4_1 ViG4_2 ViG4_3 ViG4_4

PBG4 PBG4_1 PBG4_2 PBG4_3

AHG4 AHG4_1 AHG4_2 AHG4_3

ViG7 ViG7_1 ViG7_2 ViG7_3 ViG7_4

PBG7 PBG7_1 PBG7_2 PBG7_3

AHG7 AHG7_1 AHG7_2 AHG7_3

MiG7 MiG7_1 MiG7_2 MiG7_3;

usevariables are Gender

ViG7_1 ViG7_2 ViG7_3 ViG7_4

AHG7_1 AHG7_2 AHG7_3

PBG7_1 PBG7_2 PBG7_3

MiG7_1 MiG7_2 MiG7_3;

missing all (999);

Analysis: estimator = MLR;

Model: VictL by ViG7_1* ViG7_2 ViG7_3 ViG7_4;

AdultsL by AHG7_1* AHG7_2 AHG7_3;

PeersL by PBG7_1* PBG7_2 PBG7_3;

MindL by MiG7_1* MiG7_2 MiG7_3;

!Structural regression paths

MindL on VictL AdultsL PeersL Gender;

!Fixed factor method (fix latent factor variances !to 1)

VictL@1 AdultsL@1 PeersL@1 MindL@1;

OUTPUT: modindices (all) stdyx;

**Cross-Sectional Structural Model with Victim X Peers Interaction (7^th^ Grade)**

Variable: names are Cohort School Gender

SRG4 SRG4_1 SRG4_2 SRG4_3

SRG7 SRG7_1 SRG7_2 SRG7_3

ViG4 ViG4_1 ViG4_2 ViG4_3 ViG4_4

PBG4 PBG4_1 PBG4_2 PBG4_3

AHG4 AHG4_1 AHG4_2 AHG4_3

ViG7 ViG7_1 ViG7_2 ViG7_3 ViG7_4

PBG7 PBG7_1 PBG7_2 PBG7_3

AHG7 AHG7_1 AHG7_2 AHG7_3

MiG7 MiG7_1 MiG7_2 MiG7_3;

usevariables are Gender

ViG7_1 ViG7_2 ViG7_3 ViG7_4

AHG7_1 AHG7_2 AHG7_3

PBG7_1 PBG7_2 PBG7_3

MiG7_1 MiG7_2 MiG7_3;

missing all (999);

Analysis: estimator = MLR;

Type = random;

Algorithm = integration;

Model: VictL by ViG7_1* ViG7_2 ViG7_3 ViG7_4;

AdultsL by AHG7_1* AHG7_2 AHG7_3;

PeersL by PBG7_1* PBG7_2 PBG7_3;

MindL by MiG7_1* MiG7_2 MiG7_3;

!Specify latent variable interaction

victXadults | VictL XWITH AdultsL;

!Structural regression paths

MindL on VictL AdultsL PeersL Gender victXpeers;

!Fixed factor method (fix latent factor variances !to 1)

VictL@1 AdultsL@1 PeersL@1 MindL@1;

OUTPUT: !request covariance matrix for parameter !estimates, to test simple slopes

stdyx TECH1 TECH3;

**Longitudinal Measurement Model**

Variable: names are Cohort School Gender

SRG4 SRG4_1 SRG4_2 SRG4_3

SRG7 SRG7_1 SRG7_2 SRG7_3

ViG4 ViG4_1 ViG4_2 ViG4_3 ViG4_4

PBG4 PBG4_1 PBG4_2 PBG4_3

AHG4 AHG4_1 AHG4_2 AHG4_3

ViG7 ViG7_1 ViG7_2 ViG7_3 ViG7_4

PBG7 PBG7_1 PBG7_2 PBG7_3

AHG7 AHG7_1 AHG7_2 AHG7_3

MiG7 MiG7_1 MiG7_2 MiG7_3;

usevariables are

SRG4_1 SRG4_2 SRG4_3

ViG4_1 ViG4_2 ViG4_3 ViG4_4

AHG4_1 AHG4_2 AHG4_3

PBG4_1 PBG4_2 PBG4_3

MiG7_1 MiG7_2 MiG7_3;

missing all (999);

Analysis: estimator = MLR;

Model: SRL by SRG4_1* SRG4_2 SRG4_3;

VictL by ViG4_1* ViG4_2 ViG4_3 ViG4_4;

AdultsL by AHG4_1* AHG4_2 AHG4_3;

PeersL by PBG4_1* PBG4_2 PBG4_3;

MindL by MiG7_1* MiG7_2 MiG7_3;

!Fixed factor method (fix latent factor variances !to 1)

SRL@1 VictL@1 AdultsL@1 PeersL@1 MindL@1;

OUTPUT: modindices (all) stdyx;

**Longitudinal Structural Model**

Variable: names are Cohort School Gender

SRG4 SRG4_1 SRG4_2 SRG4_3

SRG7 SRG7_1 SRG7_2 SRG7_3

ViG4 ViG4_1 ViG4_2 ViG4_3 ViG4_4

PBG4 PBG4_1 PBG4_2 PBG4_3

AHG4 AHG4_1 AHG4_2 AHG4_3

ViG7 ViG7_1 ViG7_2 ViG7_3 ViG7_4

PBG7 PBG7_1 PBG7_2 PBG7_3

AHG7 AHG7_1 AHG7_2 AHG7_3

MiG7 MiG7_1 MiG7_2 MiG7_3;

usevariables are

Gender

SRG4_1 SRG4_2 SRG4_3

ViG4_1 ViG4_2 ViG4_3 ViG4_4

AHG4_1 AHG4_2 AHG4_3

PBG4_1 PBG4_2 PBG4_3

MiG7_1 MiG7_2 MiG7_3;

missing all (999);

Analysis: estimator = MLR;

Model: SRL by SRG4_1* SRG4_2 SRG4_3;

VictL by ViG4_1* ViG4_2 ViG4_3 ViG4_4;

AdultsL by AHG4_1* AHG4_2 AHG4_3;

PeersL by PBG4_1* PBG4_2 PBG4_3;

MindL by MiG7_1* MiG7_2 MiG7_3;

!Structural regression paths

MindL on VictL AdultsL PeersL Gender SRL;

!Fixed factor method (fix latent factor variances !to 1)

SRL@1 VictL@1 AdultsL@1 PeersL@1 MindL@1;

OUTPUT: modindices (all) stdyx;

**Longitudinal Structural Model with Victim X Peers Interaction**

Variable: names are Cohort School Gender

SRG4 SRG4_1 SRG4_2 SRG4_3

SRG7 SRG7_1 SRG7_2 SRG7_3

ViG4 ViG4_1 ViG4_2 ViG4_3 ViG4_4

PBG4 PBG4_1 PBG4_2 PBG4_3

AHG4 AHG4_1 AHG4_2 AHG4_3

ViG7 ViG7_1 ViG7_2 ViG7_3 ViG7_4

PBG7 PBG7_1 PBG7_2 PBG7_3

AHG7 AHG7_1 AHG7_2 AHG7_3

MiG7 MiG7_1 MiG7_2 MiG7_3;

usevariables are

Gender

SRG4_1 SRG4_2 SRG4_3

ViG4_1 ViG4_2 ViG4_3 ViG4_4

AHG4_1 AHG4_2 AHG4_3

PBG4_1 PBG4_2 PBG4_3

MiG7_1 MiG7_2 MiG7_3;

missing all (999);

Analysis: estimator = MLR;

Type = random;

Algorithm = integration;

Model: SRL by SRG4_1* SRG4_2 SRG4_3;

VictL by ViG4_1* ViG4_2 ViG4_3 ViG4_4;

AdultsL by AHG4_1* AHG4_2 AHG4_3;

PeersL by PBG4_1* PBG4_2 PBG4_3;

MindL by MiG7_1* MiG7_2 MiG7_3;

!Specify latent variable interaction

victXpeers | VictL XWITH PeersL;

!Structural regression paths

MindL on VictL AdultsL PeersL Gender SRL victXpeers;

!Fixed factor method (fix latent factor variances !to 1)

SRL@1 VictL@1 AdultsL@1 PeersL@1 MindL@1;

OUTPUT: !request covariance matrix for parameter !estimates, to test simple slopes

stdyx TECH1 TECH3;

**Person-Centered Analyses:**

**Latent Profile Analysis with 4 Classes (4^th^ Grade)**

Variable: names are Cohort School Gender

SRG4 SRG4_1 SRG4_2 SRG4_3

SRG7 SRG7_1 SRG7_2 SRG7_3

ViG4 ViG4_1 ViG4_2 ViG4_3 ViG4_4

PBG4 PBG4_1 PBG4_2 PBG4_3

AHG4 AHG4_1 AHG4_2 AHG4_3

ViG7 ViG7_1 ViG7_2 ViG7_3 ViG7_4

PBG7 PBG7_1 PBG7_2 PBG7_3

AHG7 AHG7_1 AHG7_2 AHG7_3

MiG7 MiG7_1 MiG7_2 MiG7_3;

usevariables are ViG4 PBG4 AHG4;

missing all (999);

classes = c(4);

Analysis: TYPE = mixture;

starts = 500 100;

stiterations = 50;

LRTBOOTSTRAP = 100;

LRTSTARTS = 0 0 50 20;

k-1STARTS = 50 20;

Model:

%OVERALL%

ViG4 with PBG4 AHG4;!covariances among indicators

PBG4 with AHG4;

SAVEDATA: !save class membership for sensitivity analysis

file is LP4.txt;

save is cprob;

OUTPUT: SAMPSTAT TECH11 TECH14;

**Latent Profile Analysis – BCH Procedure Step 1**

Variable: names are Cohort School Gender

SRG4 SRG4_1 SRG4_2 SRG4_3

SRG7 SRG7_1 SRG7_2 SRG7_3

ViG4 ViG4_1 ViG4_2 ViG4_3 ViG4_4

PBG4 PBG4_1 PBG4_2 PBG4_3

AHG4 AHG4_1 AHG4_2 AHG4_3

ViG7 ViG7_1 ViG7_2 ViG7_3 ViG7_4

PBG7 PBG7_1 PBG7_2 PBG7_3

AHG7 AHG7_1 AHG7_2 AHG7_3

MiG7 MiG7_1 MiG7_2 MiG7_3;

usevariables are ViG4 AHG4 PBG4;

missing all (999);

!carry over the below variables into saved dataset !for Step 3

auxiliary are Gender SRG4 MiG7;

classes = c(4);

Analysis:

Type = mixture;

starts = 500 100;

stiterations = 50;

Model:

%OVERALL%

ViG4 with PBG4 AHG4;!covariances among indicators

PBG4 with AHG4;

Output: Tech1 svalues;

Plot: Type = plot3;

series = ViG4 AHG4 PBG4 (*);

SaveData: !carry over bch error weights into saved dataset
 !for Step 3

file is Step1BCH_4class_savedata.txt;

save = bchweights;

format = free;

MISSFLAG = 999;

**Latent Profile Analysis – BCH Procedure Step 3**

!Use dataset saved from Step 1

Data: FILE IS Step1BCH_4class_savedata.txt;

Variable: names are

!Copy/paste variable names from end of output !file from Step 1, and change "C" to "CMOD"

VIG4

AHG4

PBG4

GENDER

SRG4

MIG7

BCHW1

BCHW2

BCHW3

BCHW4

CPROB1

CPROB2

CPROB3

CPROB4

CMOD;

usevariables are bchw1-bchw4 Gender SRG4 MiG7;

missing all (999);

!incorporate bch weight variables to model error !in latent class membership

training = bchw1-bchw4 (bch);

!modal latent class (most probable class !membership)

auxiliary are CMOD;

classes = c(4);

Define: !Center self-regulation so intercepts/means are
 !interpretable

center SRG4 (grandmean);

!Center self-regulation so intercepts/means are !interpretable

center Gender (grandmean);

Analysis:

Type = mixture;

starts = 500 100;

stiterations = 50;

Model:

%OVERALL%

C on Gender SRG4;!regress latent class on cov

MiG7 on Gender SRG4;!regress mindfulness on cov

%c#1%

!estimate class-specific intercept/mean for !mindfulness

[MiG7] (dm1);

!estimate class-specific var for mindfulness

MiG7;

!estimate class-specific regression of !mindfulness on gender

MiG7 on Gender (rm1a);

!estimate class-specific regression of !mindfulness on self-regulation

MiG7 on SRG4 (rm1b);

%c#2%

[MiG7] (dm2);

MiG7;

MiG7 on Gender (rm2a);

MiG7 on SRG4 (rm2b);

%c#3%

[MiG7] (dm3);

MiG7;

MiG7 on Gender (rm3a);

MiG7 on SRG4 (rm3b);

%c#4%

[MiG7] (dm4);

MiG7;

MiG7 on Gender (rm4a);

MiG7 on SRG4 (rm4b);

Model Constraint:!new model constraints create and test !pairwise differences of each distal intercept/mean in !mindfulness, across the four classes

New(dm1v2 dm1v3 dm1v4 dm2v3 dm2v4 dm3v4);

dm1v2 = dm1 - dm2;

dm1v3 = dm1 - dm3;

dm1v4 = dm1 - dm4;

dm2v3 = dm2 - dm3;

dm2v4 = dm2 - dm4;

dm3v4 = dm3 - dm4;

Output: Tech1 svalues sampstat;

SaveData:

file is Step3BCH_4class_savedata.txt;

save = cprob;

format = free;

missflag = 999;

**Sensitivity Analysis**

Variable: names are Cohort School Gender

SRG4 SRG4_1 SRG4_2 SRG4_3

SRG7 SRG7_1 SRG7_2 SRG7_3

ViG4 ViG4_1 ViG4_2 ViG4_3 ViG4_4

PBG4 PBG4_1 PBG4_2 PBG4_3

AHG4 AHG4_1 AHG4_2 AHG4_3

ViG7 ViG7_1 ViG7_2 ViG7_3 ViG7_4

PBG7 PBG7_1 PBG7_2 PBG7_3

AHG7 AHG7_1 AHG7_2 AHG7_3

MiG7 MiG7_1 MiG7_2 MiG7_3

VictimG4_L1_v2

PeerBelG4_L1_v2

AdultHomeG4_v2

C1prob

C2prob

C3prob

C4prob

Class

UWRAdum

VSRdum

MVdum

FRdum;!dummy variable for Flourishing Rel Class

usevariables are

Gender

UWRAdum

VSRdum

MVdum

SRG4_1 SRG4_2 SRG4_3

MiG7_1 MiG7_2 MiG7_3;

missing all (999);

Analysis: estimator = MLR;

Model: SRL by SRG4_1* SRG4_2 SRG4_3;

MindL by MiG7_1* MiG7_2 MiG7_3;

!Structural regression paths

MindL on UWRAdum VSRdum MVdum Gender SRL;

!Fixed factor method (fix latent factor variances !to 1)

SRL@1 MindL@1;

OUTPUT: modindices (all) stdyx;
