# Supplementary material for: Naturalistic development of trait mindfulness: A longitudinal examination of victimization and supportive relationships in early adolescence
Source: PLoS One. 2021 May 7;16(5):e0250960. doi: 10.1371/journal.pone.0250960 (PMC8104379; doi:10.1371/journal.pone.0250960)
Supplement: Attachment — Submitted filename: Response to Reviewers.docx [file pone.0250960.s003.docx]

Response to Reviewers

Editor’s comments:

Dear Dr. Warren,

Thank you for submitting your manuscript to PLOS ONE. After careful consideration, we feel that it has merit but does not fully meet PLOS ONE’s publication criteria as it currently stands. Therefore, we invite you to submit a revised version of the manuscript that addresses the points raised during the review process.

The paper has several strengths including a large sample and longitudinal data. We were fortunate to get two experienced reviewers and I have read the paper as well. The reviewers did an excellent job describing the concerns and limitations of the paper. I will not reiterate each of their points, but rather, I will highlight several aspects of the paper that require your attention.

The paper requires major revisions, particularly regarding the data analyses. If you decide to revise and resubmit the paper, there is not guarantee that it will be accepted even with the changes.

Please provide a clearer theoretical rationale for conducting the LPA (Reviewer 1). Reviewer 2 suggested extracting the classes using grade 4 data to predict changes in mindfulness from grade 7, controlling for grade 4.

*Response: We have provided a more robust theoretical rational for our person-centered analyses in the Intro and Discussion, and after a review of literature found one relevant person-centered study that is now described in the Intro. We have also re-analyzed the data by extracting latent classes using the 4^th^ grade data to predict differences in mindfulness in 7^th^ grade, controlling for self-regulation (a proxy for mindfulness) in 4^th^ grade.*

Reviewer 2 had several important questions about the psychometrics of the mindfulness measure used. For example, what is the validity of the Interpersonal Mindfulness in Teaching Scale for use in a sample of children? What were the psychometric characteristics of the specific mindfulness facets used?

*Response: In regard to adapting items from the Interpersonal Mindfulness in Teaching Scale for use with children and early adolescents, we note in the Measures section that these items “have been used in a* *large-scale survey of elementary and high school students’ social and emotional competencies by the American Institutes for Research (AIR) and the Collaborative for Academic, Social, and Emotional Learning (CASEL)." In addition, in the Limitations section, we state that previous usage by these organizations with student samples suggest “that the mindfulness items are regarded as developmentally appropriate adaptations to the teacher measure.”*

*In regard to mindfulness facets, the Interpersonal Mindfulness in Teaching Scale has not previously differentiated specific aspects of mindfulness (we’d originally reasoned about mindfulness facets for each item in our original submission). Accordingly, we have dropped mention of any specific mindfulness facets connected to our items, and we do not comment on the psychometric characteristics of specific mindfulness facets.*

Both reviewers noted concerns about the shortened / nonstandard measures used. What is the evidence of the validity of these shortened versions of the measures? Reviewer 1 suggested conducting a factor analysis of the item level data to see if the measures are behaving as they should.

*Response:* *We have taken several steps to provide additional information concerning the quality of our measures, including: (1) reporting confirmatory factor analysis results (which were strong) from our SEM measurement models, (2) citing two prior studies that examined the construct validity of many of our constructs, (3) specifying the process by which small sets of items were selected for the larger MDI survey of which our measures are a part, and (4) creating S1 Appendix, which provides all items for all measures. A full description of these efforts can be found in our response to Reviewer 1.*

Both reviewers expressed concerns about the data analyses and suggested several alternative approaches including using an SEM framework and testing moderation through multigroup analysis (Reviewer 2). Please also address the concerns of Reviewer 1 regarding how the tests of the interactions were conducted.

*Response: The reviewers raised several critical issues that collectively informed reconceptualizing both the variable-centered and person-centered analyses. Regarding variable-centered analyses, we now employ SEM to model missing data using FIML (addressing Reviewer 2's concerns about missing data). In addition, given Reviewer 1's suggestion to examine 2-way interactions separately, we ran separate SEM models for each 2-way interaction, employing the XWITH function in Mplus to model latent variable interactions. In addition, we have taken Reviewer 1's suggestion to use LPA to extract classes based on 4th grade social ecological factors, and we used the BCH procedure to examine mean differences across latent classes in 7th grade mindfulness, controlling for 4th grade self-regulation and gender.*

Finally, please address the questions raised by Reviewer 2 about the 3-year time frame.

*Response:* *We now specify – in The Current Study portion of the Intro and in the Participants portion of the Method – that 4th and 7th grades are the two timepoints at which data were available. In addition, we now discuss the 3-year time frame in the Supportive Relationships and Mindfulness as well as the Limitations section of the Discussion.*

REVIEWER 1

Reviewer #1: The present manuscript investigates the development of mindfulness in early adolescence. Using a very large dataset (N=4593) involving 32 different schools, individuals were assessed in 4th grade and again in 7th grade. The authors found that lower victimization and greater belonging and connectedness with adults had higher levels of mindfulness years late. Person-centered analyses also identified four classes of mindfulness. The topic is very interesting, and the sample size is impressive. I offer the following critiques of the manuscript in its present form:

1) The tests of the interactions are not interpretable in their current form. When testing for a three-way interaction, it is necessary to include all underlying two-way interactions (just as seen in Table 2). However, those tests of the two-way interactions in their second step are not interpretable in this analysis. Each controls for the other, and the collinearity of XY, ZY, ZX interaction terms will be very high, and will greatly 'overcontrol' these tests. Instead, these two-way interactions should be tested in three separate models, each examining one of these two-way interactions. My guess is that this is what led to the unexpected observed effects.

*Response:* *We thank Reviewer 1 for this important suggestion. We have re-run the analyses (in SEM) and estimated each 2-way interaction in its own separate model (which also contains the main effects and covariates). Importantly, this analytic strategy did not change the presence or nature of the observed 2-way interactions, increasing our confidence that they were not a result of the analytic technique. We also note here that since we did not have hypotheses for our original (non-significant) 3-way interaction, we dropped it from our analyses.*

2) The assessment of these constructs is of course a limitation of the study because of shortened / nonstandard measures. Some of these items, though, look like they might not really measure the constructs in the study. I'm particularly thinking about the relation between the mindfulness items and the long-term self-regulation items. This would likely be a concern for most readers. I would suggest subjecting the item-level data to a factor analysis, so that it is possible to really see if these measures are behaving as they should.

*Response: We thank Reviewer 1 for raising these important points. Regarding a factor analysis of items, having now employed SEM with latent variables we report in the Results section good model fit and substantially high factor loadings for our measurement (as well as structural) models, indicating that the number of factors specified a priori was appropriate, and the content of each factor (i.e., which items load onto which factor) was appropriate, suggesting that the measures behaved as they should. Regarding the relation between mindfulness and long-term self-regulation in particular, the association was even higher at the latent variable level (r = .95) that previously observed (r = .70). Accordingly, we note in the description of the self-regulation scale that self-regulation is a constituent element in certain conceptualizations of mindfulness (Bishop et al., 2004). In addition, we have taken several steps in regard to further contextualizing our brief measures: In the Measures section we now specify that items from our shortened scales were:*

*"selected on the basis of breadth of coverage by an interdisciplinary research team with expertise in each construct, focus group feedback from children, and in consultation with key stakeholders, including educators, parents/guardians, youth program providers, and community service organizations (e.g., United Way)… The majority of the scales on the MDI were derived from existing measures of relevant constructs, and the criteria for inclusion were (1) high reliability (i.e., Cronbach’s alpha > .7) in previous research, (2) evidence of discriminant, convergent and content validity, and (3) age-appropriateness (age 9–12) of item content and wording. In order to create a survey that was both comprehensive and a manageable length to administer to students during class time, for scales with greater than three items, results of previously conducted factor analyses and reliability assessments (i.e., factor loadings, Cronbach’s alpha after an item is deleted) were used to guide data reduction in order to shorten scales to a maximum of three to five items."*

*In addition, we point to studies that have examined the construct validity of each of our brief measures except for mindfulness and long-term self-regulation, and emphasize (in the Limitations section) that the mindfulness items, while adapted from a scale for teachers, have been used with elementary and high school students by the American Institutes for Research (AIR) and the Collaborative for Academic, Social, and Emotional Learning (CASEL). We further note in the limitations section that our “mindfulness scale had adequate internal consistency and was associated with constructs within our study in expected ways.” Finally, we have provided a complete list of items for each construct in S1 Appendix so that readers have full access to this information.*

3) I wasn't really sure if the LPA analyses added much to the manuscript. It looks like classes were extracted from grade 7 data, and then associated with the grade 7 mindfulness measure. So it's really just a correlational finding. It would be more interesting if the classes were extracted using grade 4 data, and then entered into an analysis of predicting changes in mindfulness from grade 7, controlling for grade 4 mindfulness.

*Response:* *We appreciate this suggestion very much. We have re-run our person-centered analyses by using 4th grade social ecological factors as indicators for the LPA, and employing the BCH procedure to test mean differences in 7th grade mindfulness across classes, controlling for gender and 4th grade self-regulation (kindly recall that we do not have mindfulness data from 4th grade).*

REVIEWER 2

Reviewer #2: This study examined features of adolescents’ social ecologies that support or hinder the naturalistic development of trait mindfulness. Results indicated that lower victimization, higher peer belonging, and higher connectedness with adults at home predicted higher trait mindfulness between 4th and 7th grade. Moderation tests revealed mixed evidence that, contrary to hypotheses, connectedness with adults exacerbated the negative effect of peer victimization on mindfulness. Person-centered analyses yielded different categories of social ecologies, and that adolescents in “flourishing” ecologies had the highest levels of trait mindfulness. Strengths of the study include large sample size and longitudinal data collection. Below are areas of concern about the manuscript and analyses.

Introduction

The introduction did not adequately set up the latent profile analysis. Though ample space was devoted to setting up the variable-centered hypotheses, the LPA was tacked onto the end of the introduction and almost felt like an afterthought. There seem to be unique strengths associated with using a person-centered approach, in that they provide a high-level portrait of the overall social ecologies that might differentiate trait mindfulness. Are there prior studies (even with adults) that take this approach? If so, what do results reveal? The authors also suggest a practical implication for conducting LPA (for identifying adolescents who might benefit from additional support), but what are the theoretical reasons for conducting LPA?

*Response:* *We thank the reviewer very much for this suggestion. A review of literature using person-centered approaches built on social ecological indicators to predict trait mindfulness yielded only one relevant study (most person-centered analyses examined latent profiles of mindfulness facets). This study has been incorporated into the Victimization and Mindfulness section of the Intro. In addition, in the Current Study portion of the Intro we now set up our study as a 2-step study with “variable-centered and person-centered analyses that complement one another in providing a well-rounded portrait of the roles of social experiences in the development of trait mindfulness.” Finally, in the same section we point to person-centered approaches as grounded in a "whole child" approach to development (Bergman & Magnusson, 1997), according to which complex patterns of interactive factors simultaneously operate within individuals' lives. This theoretical justification is revisited in the Discussion.*

Method

The sample includes 4593 students from over 300 elementary and middle schools. To what degree is the sample representative of the overall demographics of British Columbia?

*Response: The MDI survey from which our data come is a population-level survey. All children within participating school districts take part in the MDI unless they, or their parents, opt-out. As reported, the response rate was roughly 80%. In addition, 32 public school districts in British Columbia participated in the MDI in the years during which the study took place (2013-14 and 2014-15 for G4; 2016-17 and 2017-18 for G7). These districts were geographically diverse, including both urban and rural districts. Thus, overall, the sample is fairly representative of the overall demographics of 4th and 7th graders in public schools in British Columbia. This point is now explicitly stated in the Procedure portion of the Method section.*

The authors should justify more why they assessed adolescents’ mindfulness using the Interpersonal Mindfulness in Teaching Scale (which presumably was created to measure classroom teachers) rather than validated scales (e.g., MAAS, CAMS). Is there data to suggest this scale is a valid measure of mindfulness in this sample? Have other studies used this scale to measure mindfulness in youth? Moreover, categorizing specific items into mindfulness facets (observing, non-reacting, acting with awareness) seems a bit of a stretch. Is this how the original IMTS treats these items? Also, prior research has shown that the observing facet does not function well psychometrically in non-meditating samples of adults and adolescents. Combining an item that purportedly captures “observing” with items that purportedly capture other facets (e.g., nonreactivity) could impact the scale’s validity.

*Response: We thank the reviewer for raising these important points regarding the mindfulness scale. In the description of the mindfulness measure, we now note that these items were originally included on the MDI (the larger survey project from which our data come) to assess the social and emotional learning (SEL) dimension of “self-awareness” and related constructs (e.g., mindfulness). In addition, while these items were adapted from a mindfulness scale for teachers, the items have been used in elementary and high school student samples by the American Institutes for Research (AIR) and the Collaborative for Academic, Social, and Emotional Learning (CASEL). In other words, items that had been used previously with students were selected for the MDI. In addition, in the Limitations section we state that "the mindfulness scale had adequate internal consistency and was associated with constructs within our study in expected ways."*

*In regard to mindfulness facets, the IMTS has not previously differentiated specific aspects of mindfulness (we’d originally reasoned about mindfulness facets for each item in our original submission). Accordingly, we no longer categorize specific items into mindfulness facets, and referencing specific mindfulness facets in relation to our measure has been dropped from the Measures and Limitations section.*

Along these same lines, can the authors provide further rationale for how they pick items from each of the other scales (e.g., peer belonging)? How many items did the original scales contain? Has prior work demonstrated that the shortened versions of each scale maintain construct validity? Can the authors also provide a full list of the items used in an appendix? This will help to evaluate the full coverage of each construct.

*Response: We thank Reviewer 2 for raising these important points. We have taken several steps in regard to further contextualizing our brief measures: In the Measures section we now specify that items from our shortened scales were:*

*"selected on the basis of breadth of coverage by an interdisciplinary research team with expertise in each construct, focus group feedback from children, and in consultation with key stakeholders, including educators, parents/guardians, youth program providers, and community service organizations (e.g., United Way)… The majority of the scales on the MDI were derived from existing measures of relevant constructs, and the criteria for inclusion were (1) high reliability (i.e., Cronbach’s alpha > .7) in previous research, (2) evidence of discriminant, convergent and content validity, and (3) age-appropriateness (age 9–12) of item content and wording. In order to create a survey that was both comprehensive and a manageable length to administer to students during class time, for scales with greater than three items, results of previously conducted factor analyses and reliability assessments (i.e., factor loadings, Cronbach’s alpha after an item is deleted) were used to guide data reduction in order to shorten scales to a maximum of three to five items."*

*In addition, we point to studies that have examined the construct validity of each of our brief measures except for mindfulness and long-term self-regulation (although, as noted above, we state in the Limitations that the mindfulness measure exhibited “adequate internal consistency and was associated with constructs within our study in expected ways”). Finally, we have provided a complete list of items for each construct in S1 Appendix so that readers have full access to this information.*

Results

The authors mention that 2.3% of the data were missing overall. However, the information in Table 2 suggests a higher percentage of missing data (e.g., Longitudinal analyses show N=3,945, but with a full sample of 4,593, the missing rate would be closer to 14%). Can the authors explain these discrepancies?

*Response: We thank Reviewer 2 very much for identifying this issue. Originally, while only 2.3% of data were missing overall (i.e., 2.3% of the data cells were empty), listwise deletion resulted in omitting the 14.1% of cases that had at least one missing data point on any of the variables in the longitudinal analysis. Thanks to Reviewer 2's suggestion to employ an SEM framework (and, thus, FIML), the cross-sectional analysis now omits just nine cases (0.20%) and the longitudinal analysis omits just seven cases (0.15%). However, we should also note that employing the BCH procedure in the context of LPA led to omitting a significant number of cases (10.7%), as Mplus treats covariates and outcomes as exogenous variables and applies listwise deletion. To address this issue, we ran a sensitivity analysis using a secondary approach that omitted only 1.6% of cases, and the same pattern of results was obtained. This is described in footnote 5.*

For longitudinal analyses, a stronger analytic approach would be to conduct an autoregressive, cross-lagged panel analysis using all the variables measured at each time point. Such a model would also allow for modeling covariances within time-points. As such, I would encourage the authors to also include 7th grade self-regulation as a covariate of 7th grade mindfulness (it seems right now that they only include 4th grade self-regulation). Using a SEM framework would also permit the use of full information maximum likelihood to retain missing data (see above point).

*Response: We appreciate the suggestion to use SEM very much, and we decided to do so. This has helped significantly with the missing data issue Reviewer 2 noted above. We also think employing cross-lagged panel modeling (CLPM) is an interesting suggestion, but in our view it seems unwarranted for several reasons: (1) we don't have mindfulness data in 4th grade (recall that we are using self-regulation as a proxy); (2) we don't have substantive interest in links from self-regulation in 4th grade to social ecological factors in 7th grade; and (3) CLPM does not align with our goal to test interactions (although it is technically still possible to do so).*

Table 2 should include more information, including unstandardized regression coefficients, 95% CI, and exact p-values.

*Response: We thank Reviewer 2 for encouraging more thoroughness in Table 2, which now displays the requested information.*

The authors do a nice job setting up the moderation analyses and are transparent in reporting the results (since they do not align with hypotheses). However, I might suggest exploring whether gender moderates the associations between social ecology factors and mindfulness. For example, does peer belonging predict mindfulness more strongly for one gender vs. the other? If the authors adopt a SEM framework, I might encourage them to test moderation through multi-group analysis for all moderation tests.

*Response: We appreciate this suggestion, but testing moderation by gender was not of substantive interest in our paper. Since we did not have a theoretical rationale for examining gender differences in links from social ecology to mindfulness, we decided to stick with our original approach by simply controlling for gender.*

Discussion

Can the authors explain more in the discussion, and throughout the paper, why they used a 3-year time frame? How might this timeline impact the interpretation of results? For example, are social ecologies expected to have some stability to impact mindfulness 3 years later? Or would there be intervening steps (e.g., high adult connectedness leads to greater trust and safety) that could produce higher mindfulness even in shifting social ecologies? Other limitations that might need to be acknowledged would be the use of short-form versions of scales designed to measure constructs of interest.

*Response: We now specify -- in The Current Study portion of the Intro and in the Participants portion of the Method -- that 4th and 7th grades are the only two timepoints at which data were available. In addition, we now discuss the 3-year time frame in relation to developmental process in the Supportive Relationships and Mindfulness section as well as the Limitations section of the Discussion.*
