# Supplementary material for: Naturalistic development of trait mindfulness: A longitudinal examination of victimization and supportive relationships in early adolescence
Source: PLoS One. 2021 May 7;16(5):e0250960. doi: 10.1371/journal.pone.0250960 (PMC8104379; doi:10.1371/journal.pone.0250960)
Supplement: Attachment — Submitted filename: PONE-D-20-21484R1 rev2.docx [file pone.0250960.s004.docx]

This study examined correlates of trait mindfulness in students in grades 4 and 7. Strengths of the study include a large sample, high participation rates, longitudinal design, and the use of two different data analytic approaches. The following concerns should be addressed:

1. The primary concern about this paper is how the authors frame their study and interpret their findings. All references to causal language should be removed and rather, the findings should be reframed in terms of correlations and associations rather than using terms such as “longitudinal development,” the “role” of, “foster,” “impede.” No causal conclusions can be made from these data. Only studies in which randomized controlled trials that intervene to change mindfulness can draw conclusions about causes of increases in trait mindfulness. Please remove all causal language. Several examples follow.

p. 3 “longitudinal development of trait mindfulness during early adolescence”

p. 4 “Ecologies that are likely to repeatedly enable mindful states may contribute to the development of trait mindfulness”

p. 4 “Initial evidence indicates that supportive relationships may foster trait mindfulness

[12,26], whereas toxic social experiences such as peer victimization and discrimination may

impede the development of trait mindfulness [9,12].”

P. 4 “relatively few developmental periods in which so many changes occur in a relatively short

time-span.” Birth through age five? Although the authors state that there are relatively few developmental periods, I suggest that the authors change this to indicate that this is an important time period to see changes but not that it is any more or less critical than another such period as birth to age 5.

P. 5 “understanding trait mindfulness and the factors that promote or impede its development in early adolescence can provide insights into how it can be cultivated during this critical transitional period.”

P. 5. victimization “undermines the development of mindfulness”

P. 6 “potential role of victimization in mindfulness.”

P. 6. “victimization as an impediment to the development of mindfulness”

P. 6 “constructs closely related to victimization (i.e., discrimination and rumination) converge on the premise that victimization may undermine the development of mindfulness during early adolescence.” What do you mean by “closely related?” Are these correlates or similar in terms of their meaning? They might correlate with rumination, but neither victimization nor discrimination are the same as rumination.

P. 7 “Supportive relationships are particularly impactful during early adolescence”

P. 7. Supportive relationships “foster the development of mindfulness through at least two pathways”

P. 9 “Taken together, these findings suggest that supportive relationships with family and friends not only show promise in fostering the development of mindfulness, but also in diminishing the detrimental effect of victimization on mindfulness.”

P. 9 “the roles of social experiences in the development of trait mindfulness.” This study does not tell us about the “roles” of social experiences in the “development” of trait mindfulness. Rather, it looked at correlates of change.

P. 24. “indicating a small but robust relational process by which trait mindfulness develops during early adolescence.”

P. 25. “Our findings suggest that such efforts may not only decrease victimization and thereby promote wellbeing and academic outcomes, but may also support the development of mindfulness.”

P. 26 The authors state that “there were no links from 4th grade levels of these assets to mindfulness in 7th

grade, suggesting that supportive relationships do not foster the development of mindfulness.” This is a big leap. It is not possible to conclude anything about whether supportive relationships foster the development of mindfulness. The authors should simply report that finding of a lack of a significant association without suggesting anything from this null finding.

P. 27. Regarding the unexpected significant interaction, the authors state that “the pernicious effect of victimization on mindfulness was slightly *stronger.*” Again, the results do not show that victimization had an effect on mindfulness. Rather, the significant interaction should be interpreted as showing that the association between victimization and mindfulness varied as a function of level of connectedness with adults. The authors do state this correctly on P. 22 (Figure 2) “contrary to our hypothesis, at high levels of peer belonging, the negative association between victimization and mindfulness was stronger than at low levels of peer belonging.”

P. 29. The authors’ description of the results of the person-oriented analyses are stated correctly: “functioning at the highest level across all three elements of social ecology in 4th grade was associated with higher mindfulness as they transition into early adolescence.

P. 29. Victimization – but not connectedness to adults at home or peer belonging – in 4th grade seems to be the key factor (among those examined) **explaining** mindfulness levels in 7th grade.” Victimization is related to mindfulness, but it does not explain or cause levels of mindfulness in 7^th^ grade.

P. 30. Although the discussion of anti-bullying programs is interesting, it does not follow from the findings of the current study.

P. 31. “Our findings contribute to a growing body of evidence indicating that trait mindfulness

develops as a function of ecologically normative experiences in adolescents’ everyday lives.”

A second major concern involves the measures of the constructs.

a. Three items measured most of the constructs. Although the internal consistencies were reported, more information about the psychometrics of these “measures” should be provided in this paper, even if it has been reported elsewhere.
b. Given that there are only three items per construct, all three items should be included in the description of the measures or in a table so that they can be evaluated.
c. Two of the three items on the mindfulness scale seem to be measuring impulsivity rather than mindfulness characteristics such as awareness or focusing on the present.

2. Another measurement concern is that mindfulness was not measured in 4^th^ grade. The authors state that the measure of long-term self-regulation is a pseudo proxy for the mindfulness items measured in grade 7, but it is not clear that they are justified in this assertion. Mindfulness and self-regulation are highly correlated in 7^th^ grade, but the correlations of 4^th^ grade self-regulation and 7^th^ grade mindfulness are not strong, which might indicate that they are not really the same construct. This is a major concern in this paper.

The authors assert that “self-regulation was a viable proxy” for mindfulness. This statement is not sufficiently justified.

3. Given the few findings regarding the relations among victimization, parental support, and mindfulness, the authors should provide some explanation for their finding that:

“supportive relationships intensified the adverse effect of victimization on mindfulness.”

a. The authors suggest that: “peer victimization may repeatedly prompt worries about past or future bullying, detracting from adolescents’ ability to attend to experiences emerging in the present moment.” Although this is one possible explanation, wouldn’t this also be the case for any kind of worries, not just victimization by peers? Is there something about victimization in particular that would be linked to mindfulness?
b. The logic of these potential connections makes sense, as long as it is not presented as either causal or primary. Many different types of stressors could be associated with mindfulness besides victimization. Is it victimization per se or anything that leads to rumination?
c. In the Introduction (p. 6), the authors speculate about several ways the constructs might be related, but they do not test these suggestions. For example, “rumination may account for the victimization-mindfulness link, reflecting the assertion that victimization prompts worries about past or future bullying, thereby undermining present-centered awareness [9]. Research has found that adolescents who reported higher levels of victimization tended to report higher levels of rumination [36,37], and rumination cross-sectionally and longitudinally predicted lower scores on several mindfulness facets during adolescence.”

P. 7. “in the presence of people who generally hold goodwill towards them, there is little reason to worry about how they will be treated or whether they are accepted, valued, or belong”

P. 7 “supportive relationships are unlikely to elicit anxious responses that are at odds with mindfulness.”

Although these are reasonable speculations, they might be more relevant in the discussion rather than the introduction. Did they have a measure of rumination that they could include in the analyses?

d. The authors also should consider the possibility of the direction of the association between victimization and mindfulness goes the other way. Is it possible that youth who are more mindful are less likely to be victimized by peers? The authors do note possible reverse directionality (p. 27) regarding the relation between mindfulness and positive peer relationships.

**Other points to address:**

1. p. 9. Was there a developmental reason for the two ages at which mindfulness was studied other than that these were “the two timepoints at which data were available?” “these are the only cohorts to-date for whom data were available on all study-related constructs.”
2. Explain more about the larger study and sample from which these data are a part. Make it clear that this study involved the analysis of data collected as part of another study and that the larger study was not specifically designed for this purpose.
3. P. 10. Hypothesis 2: “in the presence of higher (vs. lower) levels of peer belonging and connectedness with adults at home, the negative association between victimization and mindfulness in 7th grade will be weaker.” Will be weaker than what or as compared to what?
4. Were there any differences in the findings for the sample that used paper vs. online administration in 4^th^ grade? Also, were there differences between the results for children whose teachers read the surveys to them vs. did not?
5. Please provide all 3 items of connectedness and peer belongingness, self-regulation
6. Of the three items that measured mindfulness, the first two seem to be about impulsivity rather than mindfulness:

“When I’m upset, I notice how I am feeling before I take action.”

“When difficult situations happen, I can pause without immediately acting.”

“I am aware of how my moods affect the way I treat other people.”

7. Please included a more detailed description of the data analysis plan
8. Gender was a covariate, but did they test interactions with gender?
9. Please try to link the findings from the two different data analytic approaches.
10. Include the N for each class in Table 3
11. P. 24. “Extending theory and research on the naturalistic development of trait mindfulness during

adolescence, we cross-sectionally and longitudinally tested aspects of early adolescents’ social

ecologies in explaining naturalistic variation in trait mindfulness from 4th to 7th grade.”

Please change this first sentence. This study does not extend research on the naturalistic “development” of trait mindfulness during adolescence [4^th^ grade to 7^th^ grade is not really adolescence; maybe early adolescence]. Given that they did not measure trait mindfulness in 4^th^ grade, despite their using the measure of self-regulation as a “pseudo proxy” for mindfulness, it was not, and therefore they should reframe their language about this.
