# Supplementary material for: Naturalistic development of trait mindfulness: A longitudinal examination of victimization and supportive relationships in early adolescence
Source: PLoS One. 2021 May 7;16(5):e0250960. doi: 10.1371/journal.pone.0250960 (PMC8104379; doi:10.1371/journal.pone.0250960)
Supplement: Attachment — Submitted filename: Response to Reviewer.docx [file pone.0250960.s005.docx]

**Editor’s Comments**

Dear Dr. Warren,

Thank you for submitting your manuscript to PLOS ONE. After careful consideration, we feel that it has merit but does not fully meet PLOS ONE’s publication criteria as it currently stands. Therefore, we invite you to submit a revised version of the manuscript that addresses the points raised during the review process.

The paper has several strengths including a large sample and longitudinal data. We were fortunate to get experienced reviewers and I have read the paper as well. The reviewers did an excellent job describing the concerns and limitations of the paper.

The first reviewer was satisfied that you had addressed the concerns outlined in the first review. The second reviewer had several comments that need your attention. With some rewriting, the issues likely can be addressed. I will not reiterate Reviewer 2's comments, but I request that you address each of them in your response.

- *Response: Thank you for the opportunity to revise and resubmit our manuscript. Please find our responses to each of Reviewer 2’s comments in the pages that follow.*

The paper requires modest revisions, particularly regarding the interpretation of the findings and eliminating all language that implies causality. If you decide to revise and resubmit the paper, there is no guarantee that it will be accepted even with the changes.

- *Response: We have gone through the manuscript and changed the way we discuss our findings to ensure the appropriate correlational language is now used in all instances. In addition, in the Introduction section we have ensured that we appropriately describe previous research with correlational language, given that these studies were not RCTs, and we have prefaced certain causal conceptual arguments as “theoretical”.*

Please submit your revised manuscript by April 15, 2021. If you will need more time than this to complete your revisions, please reply to this message or contact the journal office at plosone@plos.org. When you're ready to submit your revision, log on to https://www.editorialmanager.com/pone/ and select the 'Submissions Needing Revision' folder to locateyour manuscript file.

Please include the following items when submitting your revised manuscript:

A rebuttal letter that responds to each point raised by the academic editor and reviewer(s). You should upload this letter as a separate file labeled 'Response to Reviewers'.

A marked-up copy of your manuscript that highlights changes made to the original version. You should upload this as a separate file labeled 'Revised Manuscript with Track Changes'.

An unmarked version of your revised paper without tracked changes. You should upload this as a separate file labeled 'Manuscript'.

If you would like to make changes to your financial disclosure, please include your updated statement in your cover letter.

Guidelines for resubmitting your figure files are available below the reviewer comments at the end of this letter.

Regards,

Judy Garber, Ph.D.

PLOS ONE

**Reviewer 2’s Comments**

This study examined correlates of trait mindfulness in students in grades 4 and 7. Strengths of the study include a large sample, high participation rates, longitudinal design, and the use of two different data analytic approaches. The following concerns should be addressed:

1. The primary concern about this paper is how the authors frame their study and interpret their findings. All references to causal language should be removed and rather, the findings should be reframed in terms of correlations and associations rather than using terms such as “longitudinal development,” the “role” of, “foster,” “impede.” No causal conclusions can be made from these data. Only studies in which randomized controlled trials that intervene to change mindfulness can draw conclusions about causes of increases in trait mindfulness. Please remove all causal language. Several examples follow.
   - *Response: We appreciate this feedback. We have gone through the manuscript and changed the way we discuss our findings to ensure the appropriate correlational language is now being used in all instances. In addition, in the Introduction section we have ensured that we appropriately describe previous research with correlational language, given that these studies were not RCTs, and we have prefaced certain causal conceptual arguments as “theoretical”.*

p. 3 “longitudinal development of trait mindfulness during early adolescence”

- *Response: Thank you for bringing this to our awareness. We have revised it to read, “… predicting subsequent levels of trait mindfulness 3 years later during early adolescence” (p. 3).*

p. 4 “Ecologies that are likely to repeatedly enable mindful states may contribute to the development of trait mindfulness”

- *Response: We have added the following qualification to the beginning of this sentence: “Based on the TESSERA model of personality development [14], which holds in part that regularly encountered situations repeatedly trigger states that collectively comprise a trait…” (p. 4). We hope this makes it clear that this is a conceptual argument motivating our study, rather than a causal conclusion based on ours or others’ previous research.*

p. 4 “Initial evidence indicates that supportive relationships may foster trait mindfulness

[12,26], whereas toxic social experiences such as peer victimization and discrimination may

impede the development of trait mindfulness [9,12].”

- *Response: We have revised this sentence to read, “Initial evidence indicates that supportive relationships are associated with higher levels of trait mindfulness [12,26], whereas toxic social experiences such as peer victimization and discrimination are associated with lower levels of trait mindfulness [9,12]” (p. 4).*

P. 4 “relatively few developmental periods in which so many changes occur in a relatively short

time-span.” Birth through age five? Although the authors state that there are relatively few developmental periods, I suggest that the authors change this to indicate that this is an important time period to see changes but not that it is any more or less critical than another such period as birth to age 5.

- *Response: We agree that early adolescence is not more or less critical than the birth to age 5 period. To avoid such an implication, we have revised this statement to simply read, “Indeed, rapid changes occur across an array of domains and contexts in a relatively short time-span…” (p. 5).*

P. 5 “understanding trait mindfulness and the factors that promote or impede its development in early adolescence can provide insights into how it can be cultivated during this critical transitional period.”

- *Response: We have revised this part to read, “factors associated with it in early adolescence can provide insights into how it might be cultivated...” (p. 5).*

P. 5. victimization “undermines the development of mindfulness”

- *Response: We have clarified that this is a theorized causal process proposed by previous research. The statement now reads, “… it has been theorized that victimization similarly undermines the development of mindfulness [9]” (p. 5). Hopefully, this makes clear that this is a previously-made conceptual argument that motivates our study, rather than a causal conclusion based on ours or others’ previous research.*

P. 6 “potential role of victimization in mindfulness.”

- *Response: We altered the phrase to now read, “understanding the link between victimization and mindfulness” (p. 6).*

P. 6. “victimization as an impediment to the development of mindfulness”

- *Response: We now characterize the extant empirical evidence using more circumspect language. It now reads, “… this web of empirical findings further suggests that victimization is a potential impediment to the development of mindfulness” (p. 6).*

P. 6 “constructs closely related to victimization (i.e., discrimination and rumination) converge on the premise that victimization may undermine the development of mindfulness during early adolescence.” What do you mean by “closely related?” Are these correlates or similar in terms of their meaning? They might correlate with rumination, but neither victimization nor discrimination are the same as rumination.

- *Response: Thank you for this question. We did not mean to imply that discrimination or rumination are the same as victimization. We have revised it to read, “studies examining correlates of victimization (i.e., discrimination and rumination)…” (p. 6).*

P. 7 “Supportive relationships are particularly impactful during early adolescence”

- *Response: We have replaced the term “impactful” (which we agree implies causality) with “important,” as well as added the circumspect language “seem to be.” It now reads, “Previous research indicates that supportive relationships seem to be particularly important…” (p. 7).*

P. 7. Supportive relationships “foster the development of mindfulness through at least two pathways”

- *Response: In this case we decided not to make a change. We would like to draw the reviewer’s attention to the fact that this is an argument we are proposing about developmental process, rather a causal conclusion based on ours or others’ previous research. The full sentence reads, “We argue that relationships characterized by high levels of trust, safety, and support may foster the development of mindfulness through at least two pathways” (p. 7).*

P. 9 “Taken together, these findings suggest that supportive relationships with family and friends not only show promise in fostering the development of mindfulness, but also in diminishing the detrimental effect of victimization on mindfulness.”

- *Response: We have revised this sentence to better align with the correlational nature of previous findings. It now reads, “Taken together, these findings suggest that supportive relationships with family and friends not only have longitudinal and cross-sectional links to mindfulness, but also moderate the negative association between victimization and mindfulness” (p. 9).*

P. 9 “the roles of social experiences in the development of trait mindfulness.” This study does not tell us about the “roles” of social experiences in the “development” of trait mindfulness. Rather, it looked at correlates of change.

- *Response: We have revised the phrase to now read, “well-rounded portrait of associations between social experiences and trait mindfulness” (p. 9).*

P. 24. “indicating a small but robust relational process by which trait mindfulness develops during early adolescence.”

- *Response: Thank you for raising this issue. This phrase now reads, “indicating small but robust relational correlates of trait mindfulness in early adolescence” (p. 26).*

P. 25. “Our findings suggest that such efforts may not only decrease victimization and thereby promote wellbeing and academic outcomes, but may also support the development of mindfulness.”

- *Response: In our view, the language “suggest” and “may” avoid communicating firm causal conclusions, so we have left this sentence as-is (p. 27).*

P. 26 The authors state that “there were no links from 4th grade levels of these assets to mindfulness in 7^th^ grade, suggesting that supportive relationships do not foster the development of mindfulness.” This is a big leap. It is not possible to conclude anything about whether supportive relationships foster the development of mindfulness. The authors should simply report that finding of a lack of a significant association without suggesting anything from this null finding.

- *Response: Thank you for raising this important point. We now end the sentence earlier, after reporting that “there were no links from 4th grade levels of these assets to mindfulness in 7th grade” (p. 28).*

P. 27. Regarding the unexpected significant interaction, the authors state that “the pernicious effect of victimization on mindfulness was slightly *stronger.*” Again, the results do not show that victimization had an effect on mindfulness. Rather, the significant interaction should be interpreted as showing that the association between victimization and mindfulness varied as a function of level of connectedness with adults. The authors do state this correctly on P. 22 (Figure 2) “contrary to our hypothesis, at high levels of peer belonging, the negative association between victimization and mindfulness was stronger than at low levels of peer belonging.”

- *Response: We have revised this phrase to read, “the negative association between victimization and mindfulness was slightly stronger…” (p. 29).*

P. 29. The authors’ description of the results of the person-oriented analyses are stated correctly: “functioning at the highest level across all three elements of social ecology in 4th grade was associated with higher mindfulness as they transition into early adolescence.

- *Response: Thank you.*

P. 29. Victimization – but not connectedness to adults at home or peer belonging – in 4th grade seems to be the key factor (among those examined) **explaining** mindfulness levels in 7th grade.” Victimization is related to mindfulness, but it does not explain or cause levels of mindfulness in 7^th^ grade.

- *Response: We have revised this phrase to read, “… seems to be the key factor (among those examined) associated with mindfulness levels in 7^th^ grade” (p. 32).*

P. 30. Although the discussion of anti-bullying programs is interesting, it does not follow from the findings of the current study.

- *Response: We agree. Thank you for raising this point. We have deleted the material about anti-bullying programs from what is now p. 32.*

P. 31. “Our findings contribute to a growing body of evidence indicating that trait mindfulness

develops as a function of ecologically normative experiences in adolescents’ everyday lives.”

- *Response: We have revised this phrase to read, “indicating that trait mindfulness is associated with ecologically normative experiences in adolescents’ everyday lives” (p. 33).*

A second major concern involves the measures of the constructs.

- Three items measured most of the constructs. Although the internal consistencies were reported, more information about the psychometrics of these “measures” should be provided in this paper, even if it has been reported elsewhere.
  - *Response: We have added further information on the psychometrics of all measures for which they are available. This includes the peer victimization, connectedness with adults at home, and peer belonging measures. Regarding the use of short measures, we would like to point out that the data reduction process used to shorten the scales to a maximum of three to five items as utilized in the MDI was guided by factor analyses, reliability, and validity assessments, as described in the first paragraph of the measures section on pp. 13-14.*
- Given that there are only three items per construct, all three items should be included in the description of the measures or in a table so that they can be evaluated.
  - *Response:* *All items for all constructs are provided in S1 Appendix, as per Reviewer 2’s suggestion in the first round of comments.*
- Two of the three items on the mindfulness scale seem to be measuring impulsivity rather than mindfulness characteristics such as awareness or focusing on the present.
  - *Response: Thank you for raising this point. We would like to point out that theoretical and empirical foundations of mindfulness include “nonreactivity” and the self-regulation of attention as constituent elements of mindfulness. Thus, in our view the items we used, taken from the Interpersonal Mindfulness in Teaching Scale (IMTS), are consistent with how contemplative science conceptualizes mindfulness. We also note that in an earlier version of the manuscript we conceptually linked each item to mindfulness facets (e.g., nonreactivity, acting with awareness) from the five-facet mindfulness questionnaire, but Reviewer 2 cautioned against doing so since the IMTS was not designed for that purpose. Thus, in our first revision we removed mention of mindfulness facets from the manuscript. As a compromise, in this second revision we have added the statement, “Arguably, these items capture the nonreactivity and acting with awareness aspects of mindfulness from the five-facet framework [16]” (p. 14).*

2. Another measurement concern is that mindfulness was not measured in 4^th^ grade. The authors state that the measure of long-term self-regulation is a pseudo proxy for the mindfulness items measured in grade 7, but it is not clear that they are justified in this assertion. Mindfulness and self-regulation are highly correlated in 7^th^ grade, but the correlations of 4^th^ grade self-regulation and 7^th^ grade mindfulness are not strong, which might indicate that they are not really the same construct. This is a major concern in this paper.

The authors assert that “self-regulation was a viable proxy” for mindfulness. This statement is not sufficiently justified.

- *Response: We agree that mindfulness and self-regulation are not identical constructs. However, there are strong theoretical reasons for controlling for self-regulation, which, as noted on p. 16 is a central feature of conceptual foundations of mindfulness (e.g., Bishop et al., 2004). Combined with the empirically strong association (r = .95 at the latent variable level) between mindfulness and self-regulation in 7^th^ grade, we believe that 4^th^ grade self-regulation served as a useful proxy for mindfulness. If we were to design a study from scratch to understand the development of mindfulness, we would have measured mindfulness at both timepoints, but we are making do with the data available to us.*
- *Regarding the modest correlation between 4^th^ grade self-regulation and 7^th^ grade mindfulness (r = .27), we note that the size of this correlation is not meaningfully different from self-regulation’s correlation with itself between 4^th^ and 7^th^ grades (r = .33; see Table 1). In our view, these modest associations speak to the developmental plasticity of the early adolescent period, as well as the 3-year gap during which much can change in a young person’s life.*

3. Given the few findings regarding the relations among victimization, parental support, and mindfulness, the authors should provide some explanation for their finding that:

“supportive relationships intensified the adverse effect of victimization on mindfulness.”

- *Response: We have revised this phrase to read, “we found interactions indicating that supportive relationships may intensify the negative association between victimization and mindfulness” (p. 26). This sentence is in the first paragraph of the discussion and represents part of the overview of findings. The goal is to provide a high-level summary of findings, including about the interaction effects that we observed. Since the observed interactions were very small, some interactions were not statistically significant, and the interactions that were found implicated different relational groups (peers vs. adults) in the longitudinally versus cross-sectional analyses, we added the term “may” to signify this uncertainty. More thorough discussion of the interaction effects is found on pp. 29-30.*
  - The authors suggest that: “peer victimization may repeatedly prompt worries about past or future bullying, detracting from adolescents’ ability to attend to experiences emerging in the present moment.” Although this is one possible explanation, wouldn’t this also be the case for any kind of worries, not just victimization by peers? Is there something about victimization in particular that would be linked to mindfulness?
    - *Response: Thank you for raising this question. Yes, research shows that worries (i.e., anxiety) is negatively linked to mindfulness. Our focus, however, is on aspects of children’s social ecologies that are associated with mindfulness. This shifts the focus from individual- to contextual-level factors. Peer victimization is one such relevant context, and part of the rationale for selecting this variable is that it is particularly prevalent in early adolescence (see p. 5), and it is a potent cause for worries during a time when young people’s sensitivity to peer evaluations/sanctions is heightened. A young adolescent might be worried about other issues as well (e.g., climate change), but such issues may not be front-and-center in their daily lives. Peer victimization is a highly salient part of victimized adolescents’ lives.*
  - The logic of these potential connections makes sense, as long as it is not presented as either causal or primary. Many different types of stressors could be associated with mindfulness besides victimization. Is it victimization per se or anything that leads to rumination?
    - *Response: We agree that many stressors could be associated with mindfulness, not just victimization. When considering early adolescents’ social ecologies, victimization seems to be a potent candidate, given early adolescents’ heightened sensitivity to peer evaluations/sanctions. In the Discussion we have called for future research to “examine rumination as a potential mediating pathway by which victimization undermines the development of mindfulness” (p. 27). The implication here is that victimization is not a primary/proximal cause of low levels of mindfulness; rumination might be.*
  - In the Introduction (p. 6), the authors speculate about several ways the constructs might be related, but they do not test these suggestions. For example, “rumination may account for the victimization-mindfulness link, reflecting the assertion that victimization prompts worries about past or future bullying, thereby undermining present-centered awareness [9]. Research has found that adolescents who reported higher levels of victimization tended to report higher levels of rumination [36,37], and rumination cross-sectionally and longitudinally predicted lower scores on several mindfulness facets during adolescence.”

P. 7. “in the presence of people who generally hold goodwill towards them, there is little reason to worry about how they will be treated or whether they are accepted, valued, or belong”

P. 7 “supportive relationships are unlikely to elicit anxious responses that are at odds with mindfulness.”

Although these are reasonable speculations, they might be more relevant in the discussion rather than the introduction. Did they have a measure of rumination that they could include in the analyses?

- *Response: Unfortunately, there is no measure assessing rumination in the Middle Years Development Instrument. This is certainly something we plan to examine in future research. Regarding our choice to include information about rumination in the Introduction, we believe this was a necessary part of making the case to examine peer victimization. Please note that we do return to the issue of rumination in the discussion (see pp. 27-28).*
  - The authors also should consider the possibility of the direction of the association between victimization and mindfulness goes the other way. Is it possible that youth who are more mindful are less likely to be victimized by peers? The authors do note possible reverse directionality (p. 27) regarding the relation between mindfulness and positive peer relationships.
    - *Response: Thank you for this suggestion. We have added the below sentences:* *“Future research should also consider the possibility that the link from victimization to mindfulness flows in the opposite direction. It could be the case that mindful youth are less likely to be victimized by their peers, perhaps because they are less reactive and tend to have stronger interpersonal skills” (p. 28).*

**Other points to address:**

1. p. 9. Was there a developmental reason for the two ages at which mindfulness was studied other than that these were “the two timepoints at which data were available?” “these are the only cohorts to-date for whom data were available on all study-related constructs.”
   - *Response: The MDI project from which our data are drawn focuses on 4^th^ and 7^th^ graders. The decision to survey students in these two grades was influenced by the developmental consideration that rapid changes occur across physical, cognitive, social, and emotional domains (see pp. 4-5), offering a strategic period during which to study development. The MDI was developed to capture this period by measuring children as they transition into and out of early adolescence.*
2. Explain more about the larger study and sample from which these data are a part. Make it clear that this study involved the analysis of data collected as part of another study and that the larger study was not specifically designed for this purpose.
   - *Response: We have added information about the larger MDI population monitoring project and clarify that the larger project was not specifically designed for our study. For example, we now explicitly state that, “the present study draws from data collected as part of the larger MDI population level project that monitors early adolescents’ experiences and well-being in different contexts—a project that was not specifically designed for the purposes of the current study” (pp. 11-12).*
3. P. 10. Hypothesis 2: “in the presence of higher (vs. lower) levels of peer belonging and connectedness with adults at home, the negative association between victimization and mindfulness in 7th grade will be weaker.” Will be weaker than what or as compared to what?
   - *Response: We have revised Hypothesis 2 to read, “the negative association between victimization and mindfulness in 7th grade will be weaker among those with higher (vs. lower) levels of peer belonging and connectedness with adults at home” (p. 10). We hope it is now clear that a weaker association (between victimization and mindfulness) is predicted for adolescents with higher levels of supportive relationships than for those with lower levels of supportive relationships.*
4. Were there any differences in the findings for the sample that used paper vs. online administration in 4^th^ grade? Also, were there differences between the results for children whose teachers read the surveys to them vs. did not?
   - *Response: Differential item functioning analyses have found that the MDI items do not function differently across the electronic and paper-pencil versions. Regarding teachers reading the survey aloud, we do not know which students’ teachers read the survey aloud. Rather, teachers are informed that they can read the survey aloud if they believe that their students would benefit. We have provided information on these issues on p. 13.*
5. Please provide all 3 items of connectedness and peer belongingness, self-regulation
   - *Response: All items for all constructs are provided in S1 Appendix, as per Reviewer 2’s suggestion in the first round of comments.*
6. Of the three items that measured mindfulness, the first two seem to be about impulsivity rather than mindfulness:

“When I’m upset, I notice how I am feeling before I take action.”

“When difficult situations happen, I can pause without immediately acting.”

“I am aware of how my moods affect the way I treat other people.”

- *Response: As noted above, nonreactivity and self-regulation are inherent to conceptualizations of mindfulness. We have added the below sentence to our description of the mindfulness measure: “Arguably, these items capture the nonreactivity and mindful awareness aspects of mindfulness from the five-facet framework [16]” (p. 14).*

7. Please included a more detailed description of the data analysis plan
   - *Response: We have provided additional information about the analytic plan, and believe that we now capture the analyses step-by-step in a comprehensive way. At the beginning of the section, we added that we examined missing data and computed descriptive statistics and bivariate correlations (p. 17). We also pulled information out of the footnotes – which we understand are not permitted by PLOS ONE – and now present it in the text (pp. 17 & 21). In addition, we now describe exactly which statistics we report for both the variable-centered (p. 19) and person-centered analyses (pp. 20 & 21). We have also provided information describing how data were transformed to the same 0-1 scale when reporting the final LPA solution, as well as describing that each latent class was “labeled in a manner that characterized its combination of scores across the three social ecology indicators” (p. 20). We hope these additions provide sufficient additional information regarding our analytic approach.*
8. Gender was a covariate, but did they test interactions with gender?
   - *Response: We did not originally examine moderation by gender because we did not have a theoretical rationale for doing so. To be thorough, we have since tested whether constraining the three substantive links (from social ecology predictors to mindfulness) to be invariant across genders was tenable. Constraining these paths to be equal did not result in significant loss of fit in the cross-sectional or longitudinal models, indicating that gender does not moderate the associations between the social ecology predictors and mindfulness. Accordingly, we have added the following parenthetical to the text: “(we also ruled out gender as a moderator of the substantive links by constraining the paths to be equal across genders and found that doing so did not result in significant loss in model fit)” (p. 18).*
9. Please try to link the findings from the two different data analytic approaches.
   - *Response: We would like to refer the reviewer to the following sentence: “Collectively, the person-centered findings tell a story that corroborates the variable-centered findings: Victimization – but not connectedness to adults at home or peer belonging – in 4th grade seems to be the key factor (among those examined) predicting mindfulness levels in 7th grade” (p. 32). We hope this sufficiently addresses the reviewer’s point.*
10. Include the N for each class in Table 3
    - *Response: Table 3 now presents the N for each class for every model. See p. 25.*
11. P. 24. “Extending theory and research on the naturalistic development of trait mindfulness during adolescence, we cross-sectionally and longitudinally tested aspects of early adolescents’ social ecologies in explaining naturalistic variation in trait mindfulness from 4th to 7th grade.” Please change this first sentence. This study does not extend research on the naturalistic “development” of trait mindfulness during adolescence [4^th^ grade to 7^th^ grade is not really adolescence; maybe early adolescence]. Given that they did not measure trait mindfulness in 4^th^ grade, despite their using the measure of self-regulation as a “pseudo proxy” for mindfulness, it was not, and therefore they should reframe their language about this.

- *Response: We have changed the sentence to now read, “Extending theory and research on naturalistic correlates of trait mindfulness during early adolescence, we cross-sectionally and longitudinally tested aspects of early adolescents’ social ecologies in predicting trait mindfulness levels in 7^th^ grade” (p. 26).*
